# Individualized Vibrotactile Neurofeedback Training in Patients with Chronic Bilateral Vestibulopathy

**DOI:** 10.3390/brainsci13081219

**Published:** 2023-08-18

**Authors:** Dietmar Basta, Marcos Rossi-Izquierdo, Kai Wonneberger, Cibele Brugnera, Roseli Saraiva Moreira Bittar, Mário Edvin Greters, Arne Ernst, Andrés Soto-Varela

**Affiliations:** 1 Department of Otolaryngology, Unfallkrankenhaus Berlin, University of Berlin, 12683 Berlin, Germany; 2 Department of Otolaryngology, University Hospital Lucus Augusti, 27003 Lugo, Spain; 3 Center for Otolaryngology, 47829 Krefeld, Germany; 4 Department of Otolaryngology, Faculdade de Medicina FMUSP, Universidade de Sao Paulo, Sao Paulo 01246-903, Brazil; 5 Department of Otolaryngology, Pontifícia Universidade Católica de Campinas, Campinas 13034-685, Brazil; 6 Department of Otolaryngology, Complexo Hospitalario Universitario de Santiago de Compostela, 15706 Santiago de Compostela, Spain

**Keywords:** postural imbalance, bilateral vestibulopathy, vibrotactile neurofeedback

## Abstract

Patients with bilateral vestibulopathy (BVP) suffer from postural imbalance during daily life conditions, which in turn leads to a high frequency of falls. Unfortunately, vestibular rehabilitation has only modest and somewhat inconsistent effects in this patient group. Approximately 50% of BVP patients show an improved postural control after conventional vestibular rehabilitation training. New and more promising approaches are required. The individualized vibrotactile neurofeedback training (IVNT) in stance and gait conditions has already been described as highly effective in patients with various vestibular disorders. The purpose of the present multicenter study was to determine the efficacy of the IVNT in improving balance, reducing self-perceived disability, and improving gait in patients with confirmed BVP. In total, 22 patients performed the IVNT with the Vertiguard^®^ system for 10 daily sessions. The dizziness handicap inventory (DHI), the stance stability score of the sensory organization test (SOT) and the score for everyday life mobility in stance and gait tasks (SBDT) were obtained immediately before and after the rehabilitation training period, as well as 3 and 12 months later. All measures improved significantly after the IVNT. Between 77.3% and 94.4% of patients showed an individual benefit (depending on outcome measure). The effect was not significantly reduced within the follow-up period of 12 months. The results demonstrate a high efficacy of the IVNT for vestibular rehabilitation in BVP patients.

## 1. Introduction

Bilateral vestibulopathy (BVP) is an epidemiologically rare disease. It occurs in approximately 28 of 100.000 people [1]. However, the prevalence increases with increasing age (9% in ≥65 years, 12% in ≥80 years) [2]. When those patients have subjective, clinically relevant complaints, they usually suffer from postural imbalance and unsteadiness of gait during daily life conditions that worsens in darkness and on uneven ground. This in turn leads to a high frequency of falls. A recent study reported that 43% of BVP patients experienced at least one fall within a 6-month period and 70% of them were recurrent fallers [3]. The percentage of falls in patients with BVP is significantly higher than in individuals with unilateral vestibular dysfunction [4]. Whilst 83% of patients with an uncompensated unilateral vestibulopathy (UVP) feel off-balance or unsteady, 58% of UVP patients have difficulty walking in the dark, 25% have difficulties walking on uneven surfaces, 8% have blurred vision when moving their head and 8% drift to the side when trying to walk straight, all these complaints occur in 100% of BVP patients [1]. There are typically no symptoms while sitting or lying under static conditions. Some patients also complain of oscillopsia while walking. The etiology of BVP remains largely unclear in about 50% of patients (“idiopathic”). Frequent known causes are ototoxicity (e.g., due to gentamicin) [5], bilateral Menière’s disease, autoimmune disorders, meningitis and bilateral vestibular schwannoma, as well as a combination with cerebellar degeneration (cerebellar ataxia, neuropathy, vestibular areflexia syndrome (CANVAS)) [6]. Unfortunately, in the long term, there is no improvement in vestibular function and there is currently no established causal medical treatment. The recent mainstay of treatment for patients with BVP is vestibular rehabilitation, which relies on central compensation and the reweighting of other sensory inputs [7]. Vestibular rehabilitation has been shown to be effective for numerous vestibular disorders but is less efficacious in BVP patients [8]. Vestibular rehabilitation has only modest and somewhat inconsistent effects on postural control in this patient group [9]. Approximately 50% of BVP patients show an improved postural control after conventional vestibular rehabilitation training [10,11]. There is only moderate evidence that adults with BVP improve their gait and postural stability following exercise-based vestibular rehabilitation [12]. In particular, no significant effect was found on gait speed [10,13]. This is especially important since gait speed strongly correlates with the risk of falls. It was suggested that the benefits of physical therapy are less substantial in BVP patients than in patients with other vestibular disorders because of multiple comorbidities and a slow progression in the severity of the vestibular loss [1]. Thus, the efficacy of vestibular rehabilitation in BVP patients requires improvement. There is some evidence for an increased efficiency of conventional rehabilitation, if combined with the continuous application of a noisy electrical (galvanic) stimulation (nGVS), in patients with a bilateral vestibular loss [14]. Improvements in postural control after vestibular rehabilitation tasks with nGVS may be due to an increased information throughput within the vestibular system, due to stochastic resonance.

Vestibular rehabilitation has the primary aim of promoting central compensation and, thus, improving balance function. The central compensation of vestibular deficits is essentially a physiological process, re-evaluating remaining, nonimpaired, or nonvestibular stimuli (e.g., the postural muscles, vision and proprioception) to maintain balance. Most rehabilitation programs should be started as early as possible and should include feedback mechanisms to speed up the adaptation and sensory substitution of the impaired vestibular function. The approach of vibrotactile neurofeedback training is to ensure normal posture or balance even with reduced vestibular, visual or proprioceptive input, by conveying the missing information through tactile stimulation. The vibro-tactile sense is particularly well suited to this, as it is processed intuitively and leads to an involuntary correction of posture. Furthermore, the vibrotactile stimulus, in contrast to the visual or acoustic stimulus, does not impede the acquisition of information from the environment. There are two different approaches in general: one with a permanent vibration signal depending on the body sway and a second with a signal-like stimulation only when specific swaying ranges are exceeded. The first variant is implemented, for example, by a system (BalanceBelt^®^) that is recommended for permanent wear instead of vestibular rehabilitation training. Another system (VibroTactile^®^) uses signal stimulation only if specific fluctuation values are exceeded. These thresholds are specific to each direction of body sway and should be set by a therapist. The vibratory stimulation is applied on the waist. This system is only applicable during stance tasks on a force platform. The sway is calculated based on pressure measurements on the sole of the foot. In general, the vestibular rehabilitation protocols should be individualized to provide the best possible outcome for the patients. The latest update of the Cochran Database of Systematic Reviews indicates that moderate to strong evidence exists to support vestibular rehabilitation training being applied effectively for patients with unilateral peripheral vestibular dysfunction, with the highest evidence for individualized vibrotactile neurofeedback training (IVNT) in stance and gait tasks [15].

IVNT is an approach to improve and speed up vestibular rehabilitation in stance and gait conditions, which has already been described as highly effective in patients with various vestibular disorders in randomized placebo-controlled double-blind studies. In patients with multifactorial dizziness in old age, uncompensated unilateral vestibulopathies and in Parkinson’s patients, a significant reduction in body swaying and the risk of falls has been demonstrated [16,17,18,19]. The single device with vibratory feedback, which could be used for IVNT in stance and gait tasks, is currently the Vertiguard^®^-system. The feedback thresholds are set by the system itself based on the patient’s age and sex, and the specific sensorimotor training condition.

Before the actual training, the patient completes a body sway analysis in everyday situations, with the body swaying being continuously measured close to the body’s center of gravity. This makes it possible to objectively identify and quantify the individual deficits in terms of the patient’s postural control. In the subsequent training, the patient should reduce the body sway in the conditions that are problematic with the help of the additional vibrotactile signal and thus improve the body balance in everyday life.

The purpose of the present multicenter study was to determine the efficacy of the individualized vibrotactile neurofeedback training (IVNT) in improving balance, reducing self-perceived disability, and improving gait in patients with confirmed BVP.

## 2. Materials and Methods

All patients included in this study reported dizziness and instability under daily life conditions. The total study sample included 22 participants who had chronic, uncompensated bilateral vestibulopathy. Ten female and twelve male patients with a mean age of 67.4 ± 11.3 years participated in the study.

Vestibular testing included caloric testing (horizontal semicircular canal function), recording of cervical vestibular evoked myogenic potentials (cVEMP, saccular function), and analysis of subjective visual vertical (SVV, utricular function). Diagnosis of BVP was based on the slow-phase velocity of eye nystagmus (less than 6°/s during bithermal (44 °C and 30 °C) caloric irrigation) [20]. Absent cVEMP responses were found in 40.9% and pathologic SVV-results in 27.3% of the patients. None of the participants showed severe non-vestibular sensory deficits (e.g., polyneuropathy), an acute vestibular disorder, or medication that would actively influence the vestibular system (e.g., antivertiginosa). No other treatment was provided for balance disorders during the study period.

### 2.1. Interventions

**Figure 1:**
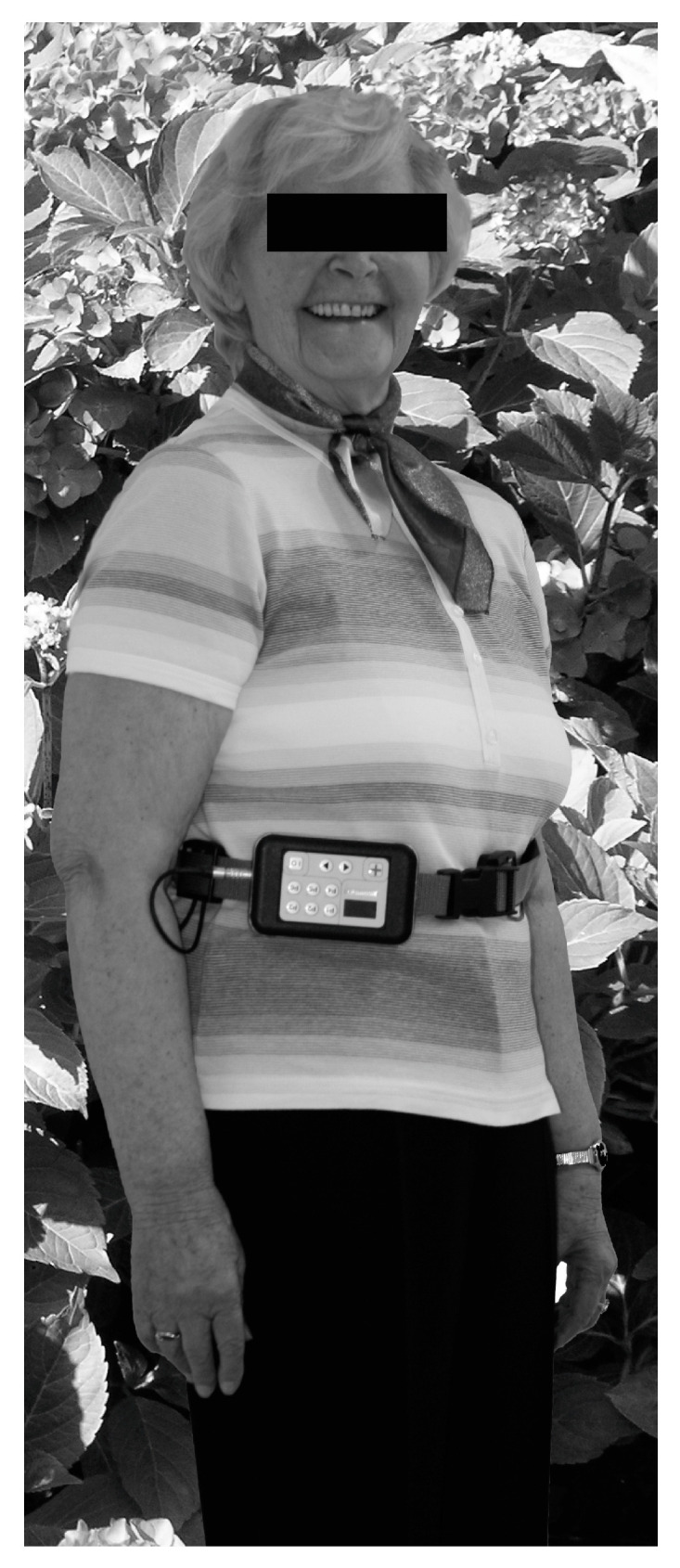
Positioning of the VertiGuard^®^-system close to the center of body mass for posturography and vibrotactile vestibular rehabilitation. Four vibratory actuators (front, back, left and right) are placed on the belt together with the main device.

Individualization of the rehabilitation program was based on a body sway analysis (mobile posturography) using the diagnostic function of the VertiGuard^®^-system (Zeisberg GmbH, Metzingen, Germany). The device was mounted with a belt at the upper pelvis (iliac crest), close to the center of mass (Figure 1). Patients younger than 60 years performed the standard balance deficit test (SBDT). All other patients performed the geriatric standard balance deficit test (gSBDT). Both tests contain a set of 14 different everyday life stance and gait conditions [16,21]. The following tasks are included in the SBDT:

- Standing on two legs with eyes open/closed;
- Standing on one leg with eyes open/closed;
- Eight tandem steps (one foot in front of the other) with eyes open;
- Standing with two legs on a foam support surface (height 10 cm; density 25 kg/m^3^) with eyes open/closed;
- Standing on one leg on a foam support surface;
- Eight tandem steps on a foam support surface;
- Walking 3 m while rotating the head;
- Walking 3 m while vertically pitching the head in rhythm;
- Walking 3 m forward with eyes open/closed;
- Walking over four barriers (height 26 cm with an inter-barrier distance of 1 m).

The tasks “standing on one leg with eyes closed” and “standing on one leg on a foam support surface” were substituted by “stand up” and “sit down” in the gSBDT.

For all stance tasks, the measurement time was 20 s and as long as required for gait tasks. The results of the body sway analysis were compared with age- and sex-related normative values. The normative values are inbuilt in the VertiGuard^®^-system and were published previously [21]. The individualized training program for vestibular rehabilitation consisted of up to six SBDT/gSBDT tasks which showed the largest positive difference from the normative values [16].

Individualized training was performed daily under supervision over 2 weeks, resulting in 10 sessions as the weekend was excluded. The feedback (rehab) mode of the VertiGuard^®^-system was used for the training. A training session consisted of five repetitions of each selected training task. Each repetition took a maximum of 20 s. During training, participants received a vibrotactile feedback signal for those directions that showed a higher body sway than preset individual thresholds. The preset threshold for each training task was related to the age and sex of the patient and could be modified in a limited range to adjust the feedback on the participant’s daily training performance. No vibrotactile feedback was applied if the participant’s sway was below a preset threshold.

### 2.2. Outcome Measures

The primary outcome measure was the Dizziness Handicap Inventory (DHI) questionnaire [22]. This questionnaire characterizes disabilities resulting from balance impairment, with scores ranging between 0 and 100. The maximum score represents the greatest disability. The DHI and all other outcome measures were obtained immediately before and after the rehabilitation training period, as well as 3 and 12 months later.

One secondary outcome measure was the SBDT/gSBDT composite score recorded without any feedback signal. The SBDT/gSBDT composite score, a risk-of-falling indicator, was calculated as the sum of ratios of all SBDT/gSBDT task scores to their age- and sex-related normative values in anterior/posterior and lateral directions. It was calculated by using the following formula:

$$SBDT/gSBDTcompositescore=\frac{(\sum_{i}pi+\sum_{i}ri)\;\cdot \;100}{n\;\cdot \;400}$$

with:

*p* = pitch sway divided by normal value in %.

*r* = roll sway divided by normal value in %.

*n* = number of tasks in the SBDT or gSBDT.

This score is scaled between 0 and 100, where 100 represents the highest risk of falling and thus represents the lowest stability [21].

Furthermore, participants underwent the sensory organization test (SOT) on the ankle-sway referenced platform BalanceMaster^®^ (Nicolet Biomedical^®^, Clackamas, OR, USA), as an additional secondary outcome measure for stance stability under different sensorimotor conditions. Measurements were taken during three repeated 20 s runs under six sensorimotor standing conditions [23]. The following stance tasks were performed in the SOT: standing with eyes open/closed, standing with a moving surrounding, standing on a tilting platform with eyes open/closed, standing on a tilting platform with a moving surrounding. The SOT composite score is scored between 0 and 100, with the highest score indicating maximal stability.

### 2.3. Statistical Analysis

Pre- and post-training values of all outcome measures were compared using the *t*-test for dependent samples if they were normally distributed, whereas for non-normally distributed data, the Wilcoxon’s test was used. The Kolmogorov–Smirnov test was chosen for testing the data distribution. The level for significance of all tests was a *p* value less than 0.05. A similar procedure was applied for the data analysis of follow-up results. Since not all patients showed up for follow-up measures, the comparisons (pre, post, 3 months, 12 months) were only performed with results from patients who participated on all visits. A Bonferroni alpha-correction was applied for multiple comparisons.

A clinically relevant change could be surely assumed if the SBDT- or SOT-score increased or decreased by 10 points [21,23] or the DHI-score increased or decreased by 18 points [24], since these values are the largest differences between qualitative interpretations.

## 3. Results

### 3.1. Comparison of the Pre-Post Rehabilitation Measures

**Figure 2:**
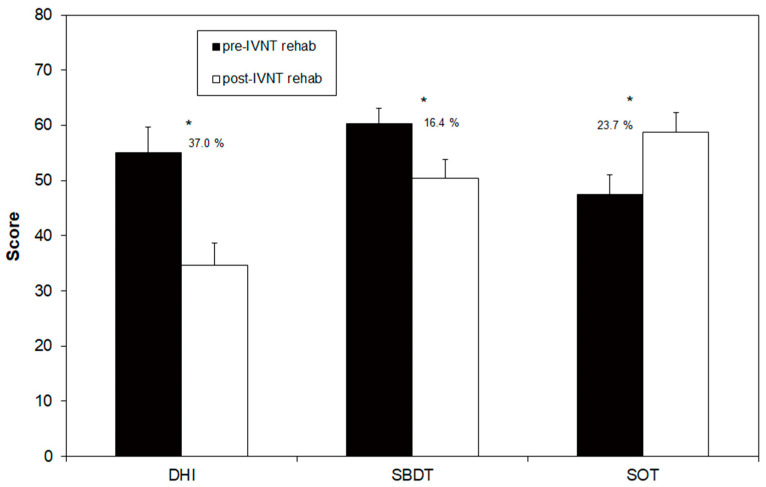
Scores of the Dizziness Handicap Inventory (DHI), the Standard Balance Deficit Test (SBDT) and the Sensory Organization Test (SOT) before and after the individualized vibrotactile neurofeedback training (IVNT). Asterisks indicate a significant difference between pre- and post-rehab values.

Objective parameters such as SBDT and SOT scores, as well as subjective parameters such as DHI scores, were calculated before and after the training period (Figure 2).

The SBDT composite score before the training was 60.3 (±3.5), which decreased to 50.4 (±3.6) after the training. This improvement of 16.4% was statistically significant and17 out of 22 patients showed a reduction in the score. A significant improvement in the SOT composite score was found when comparing pre- and post-training results: 47.5 (±2.8) and 58.8 (±3.5), respectively. The percentage increase in stance stability was 23.7%. Only one tested patient showed no improvement. DHI scores following the training were decreased: 55.1 (±4.6) pre-training to 34.7 (±4.0) post-training, with this change representing a statistically significant improvement of 37% (Figure 2). In total, 19 out of 22 patients showed a reduction in this primary outcome measure due to the IVNT. Only three patients showed nearly no change in the DHI-score after the training (Table 1).

### 3.2. Follow-Up

**Figure 3:**
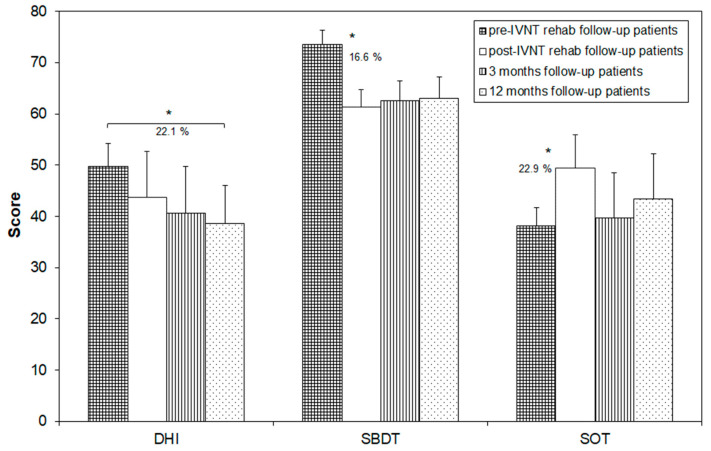
Scores of the Dizziness Handicap Inventory (DHI), the Standard Balance Deficit Test (SBDT) and the Sensory Organization Test (SOT) in patients which showed-up for the follow-up measures before and after the individualized vibrotactile neurofeedback training (IVNT) as well as 3 and 12 months later. Asterisks indicate a significant difference between the time points.

Only ten patients participated in the 3- and 12-month visits. The data of all visits were analyzed for these patients separately (Figure 3). The SBDT composite score significantly decreased from 73.5 (±6.5) to 61.3 (±9.2) after the training (16.6% change) and remained stable during the next 12 months (62.5 ± 8.8 after 3 months and 63.0 ± 9.7 after 12 months).

The SOT-score also showed a significant change from 38.2 ± 3.3 to 49.5 ± 4.9 after the training (22.9% change). There was a clear but not significant reduction in the score 3 and 12 months after the training (39.7 ± 4.0 after 3 months and 43.3 ± 4.2 after 12 months).

The DHI-score was not significantly changed, if compared before and after the training (49.7 ± 8.9 before and 43.7 ± 7.3 after the training), after the training and 3 months later (40.7 ± 9.1) and between the next 9 months of the follow-up (38.7 ± 7.4). There was a statistically significant decrease in the DHI-score by 22.1% between the pre-training values and the 12-month follow-up (Figure 3).

## 4. Discussion

A vestibular rehabilitation by IVNT over 10 days was able to significantly enhance the objectively determined postural control during stance and gait tasks. These improvements were found in both objective methods: the ankle sway referenced platform system and the sway measurement close to the center of gravity. Interestingly, not only significant group improvements were found. A total of 77.3% of all patients enhanced their postural control on an individual basis during everyday life stance and gait tasks and 94.4% during different sensorimotor stance tasks. These values are much more pronounced than earlier reported for any other vestibular rehabilitation in BVP patients. Gillespie and Minor (1999) [10] reported 51% of patients showed improvement after a conventional vestibular rehabilitation and Herdmann et al. (2015) [9] reported between 38 and 86% (depending on the outcome measure). Interestingly, the latter study showed the highest success rate in functional tests (e.g., gait speed, dynamic visual acuity) and the lowest success rate in subjective scores. However, the increased stability of the patients in the present study was also reflected by a significant decrease in the subjectively reported dizziness handicap. This holds true as a group measure as well as on an individual basis. An individual decrease in the DHI-score was observed in 86.4% of all treated patients. This is a higher rate of improvement compared to other studies. A recent study, which combined conventional vestibular training with noisy galvanic vestibular stimulation for treatment of bilateral vestibulopathy, failed to show a decrease in the DHI group value, even if the postural stability during stance tasks could be significantly improved [25]. Brown et al. (2001) [11] differentiated between the percentage of patients with a DHI improvement and the percentage of patients with a clinically significant change in the DHI score. The minimal clinically significant change was defined as a change of 18 points. Unfortunately, the background of this cut-off value was not further explained but is possibly related to the largest difference between qualitative interpretations [24]. Based on this criterion, 33% of all patients in the study of Brown et al. (2001) [11] showed a clinically significant change of the DHI scores. In the present study, the rate of improvement as calculated due to Brown et al. (2001) [11] was 54%. This evidently demonstrates the superior efficacy of the IVNT as vestibular rehabilitation measure in BVP patients.

The follow-ups could only be performed in nearly half of the patients. These patients showed no significant group improvement in the subjective measure (DHI) directly after the training and 3 months later. Possibly, this is why they participated in the follow-up visits. The phenomenon, that mainly patients with subjective poor improvement show up for follow-up visits is well documented [16]. Surprisingly, these patients enhanced their objectively measured postural control in the present study directly after the IVNT. A significant increase of the subjective improvement was only found 12 months after the training, even if the objective measures for postural control were nearly unchanged or not significantly worse meanwhile. These opposite results are possibly related to the patients’ expectations. The postural control was lower in these patients before the training, if compared to the entire group, and the improvement directly after the training was “only” similar. Since the DHI-scoring is not linearly related to the patient’s handicap (DHI; 16–34 points = mild handicap, 36–52 points = moderate handicap, >54 points = severe handicap) [24], the next step of improved handicap perception was possibly not fulfilled directly after the training.

Anyway, all follow-up measures improved after the IVNT for at least 12 months, which would suggest that such a long-term effect is not directly related to the IVNT training alone. The patients were probably mobilized and left their sedentary lifestyle since they were better able to maintain postural control during any physical activity. Thus, enhanced physical activity should have contributed to the reported long-term benefit as well.

One limitation of the study is the lack of a control group. Adding a control group is always difficult in a study that investigates a rare disease. This was also shown in previous studies by the very limited numbers of patients in the experimental group and the controls (mainly below 10) [12]. This is a disadvantage for the statistical power of the study. However, the absolute lack of any effect of a placebo application in the IVNT method (only performing the exercises without the vibrotactile stimuli) was already shown for a couple of other vestibular disorders [16]. Even if similar results are expected for BVP patients, future larger studies should include a control group (e.g., placebo).

## 5. Conclusions

The results of the current study show that BVP patients can be effectively rehabilitated with the easy to perform IVNT method in stance and gait conditions. This holds true not only for the whole investigated sample, but also for almost all individual patients. One limitation of the method is that the patient should be able to perform at least some of the tasks included in the SBDT/gSBDT. This is analyzed by the body sway analysis, which always precedes the rehabilitation training. 

Furthermore, there is some evidence that the single 10-day training could have a long-term effect that lasts at least 12 months. However, this should be further investigated in a larger sample of BVP patients with a clear record of individual physical activity.

## Figures and Tables

**Table 1:** Individual scores of the Dizziness Handicap Inventory before and after the individualized vibrotactile neurofeedback training (IVNT) and the related difference (post- minus pre-scores).

Patient	DHI Pre-IVNT	DHI Post-IVNT	Delta DHI Post-Pre
1	70	44	−26
2	96	76	−20
3	72	62	−10
4	28	36	8
5	22	24	2
6	52	28	−24
7	76	26	−50
8	74	24	−50
9	24	12	−12
10	64	28	−36
11	42	22	−20
12	26	14	−12
13	54	41	−13
14	42	16	−26
15	28	24	−4
16	58	40	−18
17	62	16	−46
18	52	18	−34
19	72	60	−12
20	64	68	4
21	42	28	−14
22	92	56	−36

## Data Availability

All data are available upon request from the corresponding author.
